# The “Duckweed Dip”: Aquatic *Spirodela polyrhiza* Plants Can Efficiently Uptake Dissolved, DNA-Wrapped Carbon Nanotubes from Their Environment for Transient Gene Expression

**DOI:** 10.1021/acssynbio.3c00620

**Published:** 2023-12-21

**Authors:** Tasmia Islam, Swapna Kalkar, Rachel Tinker-Kulberg, Tetyana Ignatova, Eric A. Josephs

**Affiliations:** Department of Nanoscience, University of North Carolina at Greensboro, 2907 E. Gate City Blvd., Greensboro, North Carolina 27401, United States

**Keywords:** duckweed, *Lemnaceae*, *Spirodela polyrhiza*, carbon nanotubes, transient expression

## Abstract

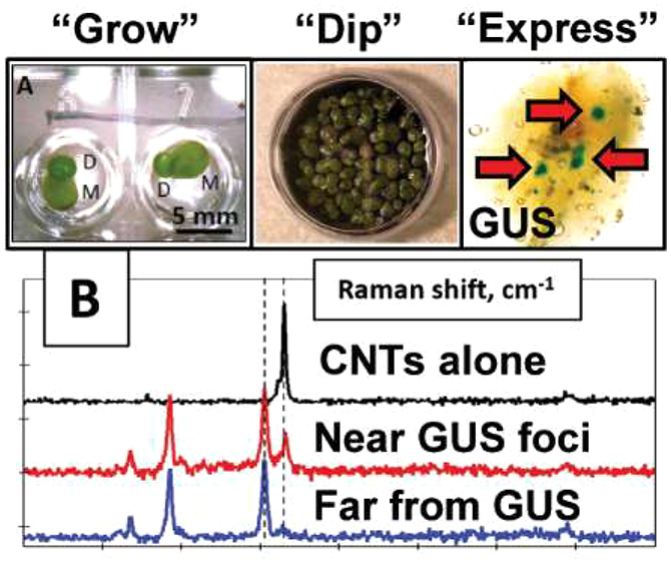

Duckweeds (*Lemnaceae*) are aquatic nongrass monocots that are the smallest and fastest-growing flowering plants in the world. While having simplified morphologies, relatively small genomes, and many other ideal traits for emerging applications in plant biotechnology, duckweeds have been largely overlooked in this era of synthetic biology. Here, we report that Greater Duckweed (*Spirodela polyrhiza*), when simply incubated in a solution containing plasmid-wrapped carbon nanotubes (DNA-CNTs), can directly uptake the DNA-CNTs from their growth media with high efficiency and that transgenes encoded within the plasmids are expressed by the plants—without the usual need for large doses of nanomaterials or agrobacterium to be directly infiltrated into plant tissue. This process, called the “duckweed dip”, represents a streamlined, “hands-off” tool for transgene delivery to a higher plant that we expect will enhance the throughput of duckweed engineering and help to realize duckweed’s potential as a powerhouse for plant synthetic biology.

Duckweeds (*Lemnaceae* or *Araceae*; there are taxonomic disagreements) are small, aquatic, nongrass monocots of which there are 37 species in five genera.^1^ They are the smallest flowering plants in the world, with the largest duckweeds (Greater Duckweed, *Spirodela polyrhiza*) consisting mostly of a small leaf-like structure known as a frond measuring <1 cm across (Figure 1A).^2^*S. polyrhiza* also has several rhizoid structures underneath their fronds, hence its scientific name.^3,4^ In addition to their simplified morphologies, they also have a significantly reduced genome with fewer than 20,000 genes; several draft genomes and transcriptomic sequencing results have been published.^5−11^ While they are capable of flowering under certain conditions, in general duckweeds survive in a juvenile state, and *S. polyrhiza* primarily propagates vegetatively by continuously budding off clonal “daughter” plantlets from two meristematic pads asexually every 1–3 days (Figure 1A).^5,12^ Their simplicity to culture in large numbers (with exponential population growth of genetically identical plants) and their ability to efficiently extract material like radiolabeled metabolites from their aquatic environments made them ideal as a model higher plant in the decades before *Arabidopsis*,^13^ but duckweeds have largely been overlooked in this emerging era of synthetic biology, where new approaches have the power to truly unleash their immense biotechnological potential.

**Figure 1:**
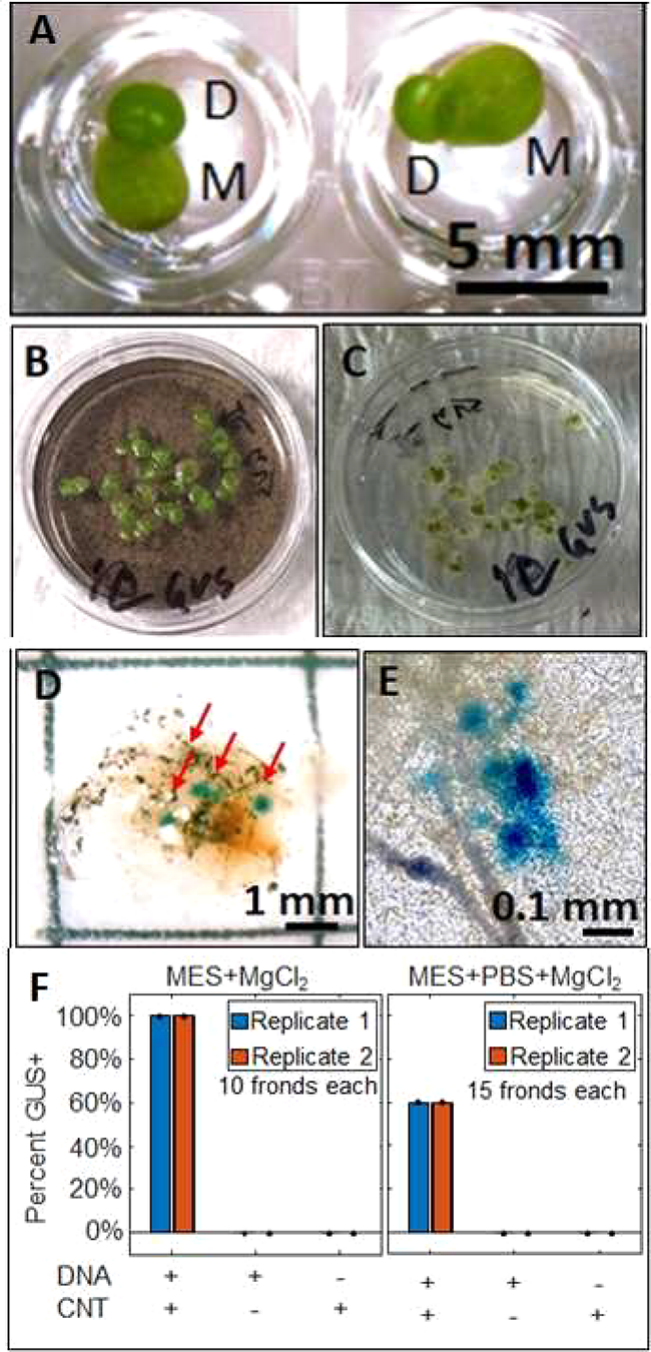
The “duckweed dip”. (A) *Spirodela polyrhiza* mother (M) and daughter (D) fronds growing in the wells of a 96-well plate. Note that while the duckweed may resemble single-celled organisms dividing, they are in fact (multicellular) higher plants, and these experiments are performed in whole plants rather than isolated cells. (B) *S. polyrhiza* plants are incubated in a 30 mm Petri dish containing a solution with carbon nanotubes (DNA-CNTs) wrapped with a plasmid containing the gene for reporter protein β-glucuronidase (GUS) under two 35S promoters. (C) After a rinsing/solution exchange to growth media. (D) 2 days after initial incubation in DNA-CNT solution, replacement of media and growth for 3 days, and GUS staining, blue foci on the fronds indicate that *S. polyrhiza* are positive for in planta GUS expression and activity. (E) Close-up of positive GUS stains in the *S. polyrhiza* fronds. (All images were brightened 20% for clarity.) (F) Effects of DNA-CNT delivery buffer (either 25 mM MES and 15 mM MgCl_2_ (MES delivery buffer); or MES delivery buffer with 0.1× PBS) on the number of fronds scored to have at least one GUS focal spot on their frond; *n* = 10 and 15 for each replicate, respectively.

With regards to their biotechnological potential, engineered *Spirodela* species can exhibit very high levels of transgene protein expression: in one report stable, transgenic, GFP yield was >25% of total soluble protein,^14,15^ among the highest in all plant expression systems. Unlike many terrestrial plants, duckweeds can also be sterilized and grown aseptically,^2^ a useful attribute for biopharmaceutical production, and engineered duckweeds have been used to generate monoclonal antibodies and pharmaceutical proteins such as anticoagulants and interferons.^15^ Duckweeds are also edible,^16^ with excellent nutritional properties and biomass accumulation rates comparable to the fastest-growing terrestrial crop plants; engineered duckweeds expressing viral antigens have been used for oral vaccination of animals when given as feed.^17−19^ A particularly powerful recent example of the potential of duckweed synthetic biology was the recent engineering of duckweed *Lemna japonica* for biofuel production:^20^ after inserting a synthetic gene cassette for estradiol-inducible cyan fluorescent protein-*Arabidopsis* WRINKLED1 fusion protein, strong constitutive expression of a mouse diacylglycerol:acyl-CoA acyltransferase 2, and a variant of sesame oleosin, the duckweed exhibited an 108-fold increase in triacylglycerol accumulation that, if grown at scale (on wastewater tracks, for example), would produce 7 times as much oil/biofuel as soy per acre and about as much as oil palm. Applications such as these motivate our work to identify simple, high-efficiency methods to manipulate duckweed biology.

The ability to rapidly screen synthetic gene constructs and cassettes can help to drive success in applications of plant synthetic biology, with duckweed in particular, by rapidly screening promoter strengths^21^ or evaluating transgene activity, silencing, or interactions with the host.^22^ This can be performed in plants using transient expression systems via agrobacterium-mediated infiltration of whole plants, including for *S. polyrhiza*.^23^ However, agrobacterium-mediated approaches tend to have low success rates (in *S. polyrhiza*(24)), can exhibit plant toxicity, and require delicate handling of the duckweeds, including removal of the fragile plantlets from water onto solid media and back again.^23,24^ In terrestrial plants, an alternative strategy for gene delivery that uses DNA-wrapped carbon nanotubes (CNTs) can also be an effective method to deliver plasmid DNA for transient expression.^25,26^ In terrestrial plants, these methods typically require manual infiltration of the nanotubes into plant tissue by wounding and injection with a blunt-tipped syringe, which would be difficult in the case of duckweed given its small size and fragility. We hypothesized that we could develop a system whereby simply incubating duckweeds with the DNA-wrapped single-wall CNTs (DNA-CNTs) would result in successful gene delivery, because they are so good at extracting material from their aquatic environments. Such a method would allow for streamlined, high-throughput transient gene expression in duckweeds with minimal “hands-on” time.

In this technical report, we first report that, after making some modifications to the reported methods to prepare DNA-loaded SWCNTs that were infiltrated into terrestrial plant tissue, *S. polyrhiza* could survive and were healthy after culture in media containing DNA-CNTs for prescribed times from 30 min to at least 48 h, followed by media exchange/rinsing with water and culture in 0.5× Schenk and Hildebrandt (SH) media for 3 days (Figures 1B,C). We then prepared DNA-CNTs (see Methods in Supporting Information) by mixing carboxylic acid-functionalized single-wall carbon nanotubes (CNTs; 4 nm diameter, with lengths <1000 nm; Figure S1) with high molecular-weight polyethylenimine (PEI) then with a ∼9000 bp plasmid with a ∼2500 bp insert under two cauliflower mosaic virus (CaMV) 35S promoters that contain 3 exons derived from the gene for β-glucuronidase (GUS) interspersed with two introns so that it can only be expressed if proper mRNA splicing occurs in plants.^27^ 100% of *S. polyrhiza* plants (at least 10 plants across two technical replicates) were positive for GUS activity straining on their fronds after 2 days incubation in delivery buffer (25 mM 2-(*N*-morpholino)ethanesulfonic acid and 15 mM MgCl_2_, MES delivery buffer) containing DNA-CNTs followed by 3 days in 0.5× SH media with cefotaxime (50 μg/mL) (Figures 1D–F and S2). We did note some sensitivity to the salts in the DNA-CNT delivery buffer, which resulted in slightly lower transformation efficiencies if the buffers had higher salt concentrations like as those used during infiltration of DNA-CNTs into the tissue of terrestrial plants (Figure 1F).^25,26^ GUS activity was not detected in plants incubated with plasmid DNA alone (or CNTs alone; Figures 1F and S3) and required gentle wounding of the plants with a thin gauge (26G) syringe needle prior to incubation, which did not appear to affect plant health.

Plants incubated for the 5 days and then washed were also imaged after GUS staining using a confocal Raman microscope,^28−32^ and the strong G-band Raman signature of SWCNTs at 1589 cm^–1^ was detected in frond tissue (Figure 2A–C); no Raman signature of SWCNTs was found in the rhizoid structures (Figure 2D). Thus, we conclude that single-walled CNTs facilitate the passive introduction of plasmid DNA into *S. polyrhiza* fronds, where transgenes can be transiently expressed. Detailed methods for this “duckweed dip” protocol, where *S. polyrhiza* are simply incubated in solutions of DNA-loaded SWCNTs followed by media exchange, and which we name in analogy to the simple “floral dip” protocol for transgene delivery used in more easily transformable higher plants like model *Arabidopsis thaliana*.^33^

**Figure 2:**
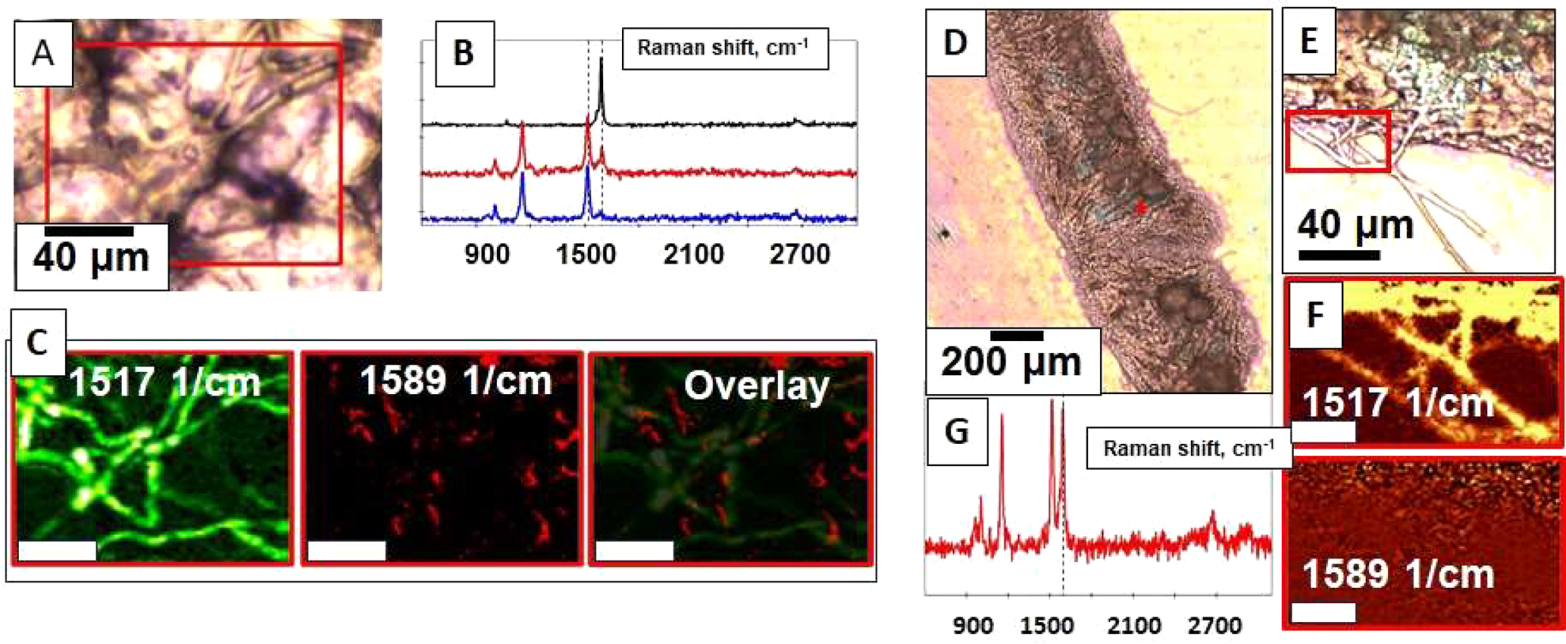
Internalization of functionalized CNTs inside of *S. polyrhiza*. (A,D,E) Optical micrographs of duckweed fronds after 2 days incubation in DNA-CNT solution and 3 days in growth media. (B) The Raman spectra showing (black) CNTs alone, (red) duckweed fronds after incubation with DNA-CNTs at the site of blue GUS staining (blue) signal from duckweed tissue away from the sites of blue GUS staining. (C) Confocal Raman images of (left) duckweed conjugated pigment structures (likely chlorophyll^34^), (middle) CNTs, and (right) overlaid Raman map. (G) Raman spectrum taken at the red star location in the (D) panel shows colocalization of CNT and the region of blue positive GUS staining. (F) On the contrast, no CNTs are seen in the Raman map of rhizoids (taken in the area highlighted by the red square in panel E). Scale in bars (C) and (F) are 10 μm.

Most organisms, particularly those not now considered model organisms, tend to be very resistant to the introduction of foreign DNA and employ a variety of methods to recognize and degrade any foreign DNA that is introduced into their cells before any genes on them can be expressed. It is notable that *S. polyrhiza* can not only uptake plasmid-wrapped CNTs but also then express genes encoded on that DNA without requiring the usual direct delivery of large doses of *Agrobacterium tumefaciens* or DNA-loaded nanomaterials directly into plant tissue. While there had been previous reports of transient expression using agrobacterium-mediated approaches in *S. polyrhiza*,^24^ the phenomenon reported here appears to be a significantly more efficient method for transient gene expression. The presence of the introns in the GUS gene strongly suggests that the plasmid DNA is being transcribed, the resulting RNA is processed and spliced properly, and transgenes are expressed within the *S. polyrhiza* fronds.

Duckweeds have significantly reduced morphologies and are among the fastest growing plant species; because they grow so fast, they are very well equipped to extract materials efficiently from their aquatic environments to facilitate that growth. However, at the moment, we do not speculate about the transport mechanism of the DNA-CNTs into the plant tissue where the transgenes they carry can be expressed, although further investigation into the nature of the DNA-CNT delivery and plasmid release is clearly of interest. We also note that immune signaling components found in duckweed are highly divergent from many other (terrestrial) plants,^35^ and perhaps these differences play a role in the ability of duckweed to uptake and express foreign genes delivered via CNTs. For these reasons, one might expect that this approach could be suitable for other biotechnologically important duckweed species as well, such as those in the *Lemna* family.

The advantages of our approach reported here are the minimal hands-on time—potentially allowing automation of the transformation process, since only buffer exchanges and gentle wounding are necessary, and the small volumes of materials required for transforming the small plants, which can be performed in 96-well plates (Figure 1). Both of those features can greatly facilitate high-throughput duckweed biology and biotechnology optimization. For example, efficient delivery of proviral replicons^24^ without the need for manual infiltration or recovery after agroinfiltration, along with automated phenotyping, would allow in principle for many simultaneous viral induced gene silencing (VIGS) experiments to be performed easily and simultaneously. We expect this simple approach to transgene delivery will allow for more efficient duckweed engineering and can serve as a useful tool to help realize duckweed’s strong potential as a powerhouse for plant synthetic biology.

## Supporting Information Available

Detailed methods, AFM and SEM images of CNTs, and additional plant images (PDF)
